# Chlorogenic Acid, the Main Antioxidant in Coffee, Reduces Radiation-Induced Apoptosis and DNA Damage via NF-E2-Related Factor 2 (Nrf2) Activation in Hepatocellular Carcinoma

**DOI:** 10.1155/2022/4566949

**Published:** 2022-08-02

**Authors:** Xin Yin, Xingkang He, Lingyun Wu, Danfang Yan, Senxiang Yan

**Affiliations:** ^1^Department of Radiation Oncology, The First Affiliated Hospital, School of Medicine, Zhejiang University, 79 Qingchun Road, Hangzhou, Zhejiang 310003, China; ^2^Department of Gastroenterology, Sir Run Run Shaw Hospital, Zhejiang University Medical School, Hangzhou 310016, China

## Abstract

Radiotherapy produces excessive reactive oxygen species (ROS), which can lead to DNA damage and apoptosis in tumor cells, thereby killing malignant cells. Chlorogenic acid (CGA) is a well-known antioxidant in coffee due to its strong ability to remove ROS. However, the effect of CGA on radiotherapeutic efficacy remains unclear. In this study, we showed that CGA could hinder the therapeutic effect of radiotherapy by inhibiting radiation-induced apoptosis and DNA damage via scavenging excessive ROS and activating the NF-E2-related factor 2 (Nrf2) antioxidant system in hepatocellular carcinoma (HCC) cells and a murine model. The knockdown of Nrf2 reversed CGA-mediated radiation resistance in HCC cells. In conclusion, CGA might be a potential tumor-protective compound upon irradiation and reduce the efficacy of radiotherapy via ROS scavenging and Nrf2 activation.

## 1. Introduction

Radiotherapy (RT) is a widely used modality in cancer treatment, applied in more than 50% of all patients with cancer with a cure rate of 40% [1, 2]. However, the complete responses of RT remain only 15% in hepatocellular carcinoma (HCC) [3]. RT resistance has always been an issue in HCC treatment, leading to a high mortality rate of HCC, especially in developing countries [3].

RT exerts its cytotoxicity mainly through the production of reactive oxygen species (ROS). The intracellular antioxidant system is closely related to the outcome of RT. This system is activated by the binding of master transcription factor NF-E2-related factor 2 (Nrf2) to the antioxidant response element of a series of cytoprotective genes, including heme oxygenase-1, NAD(P)H:quinone oxidoreductase-1, glutaredoxin 1, and thioredoxin 1 [4–8]. All of these enzymes are characterized by their ability to reverse oxidative damage and stress. Drugs or compounds that amplify the Nrf2 system are considered potential defenders of various endogenous and exogenous stresses such as hypoxia, inflammation, ultraviolet irradiation, and ionizing radiation [9–13]. Recent studies have found that Nrf2 activation by Se-hormetic agents promoted hematopoietic progenitor cell regeneration and increased the survival of irradiated mice following exposure to high doses of radiation [14, 15].

Chlorogenic acid (CGA) is one of the most abundant phenolic acid compounds in coffee and tea. It has been reported to have robust antioxidant activity [16–18] as well as many other pharmacological activities, such as anti-inflammatory [17], antidiabetic [19], hepatoprotective [20], and antitumor [21], in many preclinical and clinical studies. It is currently under clinical investigation to prevent diabetes, dyslipidemia, metabolic syndrome, endothelial dysfunction, and overweight, and treatment of solid tumors. Besides, many previous studies have demonstrated significant cytoprotective effects of CGA attributed to the activation of the Nrf2 pathway [18, 22–25].

However, the role of CGA in tumor radiotherapy remains unknown. The present study found that CGA hindered the therapeutic effect of radiotherapy in HCC both in vitro and in vivo. Specifically, CGA ameliorated RT-induced cell death, DNA damage, and apoptosis by scavenging excessive ROS and activating Nrf2 and its downstream protective genes in HCC.

## 2. Materials and Methods

### 2.1. Materials and Cell Culture

The chlorogenic acid was dissolved >98% pure (Sigma, C3878). Antibodies below used were listed in supplemental materials (available here).

Huh7, Hep3B, LO2, and Hep1-6 cell lines were obtained from Shanghai Institutes for Biological Science, China. Cells were cultured in high-glucose DMEM (Invitrogen, CA, USA), blent with 10% fetal bovine serum (Invitrogen, CA, USA), 100 *μ*g/ml streptomycin, and 100 U/ml of penicillin. The tumor cell culture dishes were placed in the humid incubator with 5% CO_2_ and 95% air at 37 °C. Experiments were performed on cells of passage less than 15.

### 2.2. Treatment with Chlorogenic Acid

CGA was dissolved in dimethyl sulphoxide (DMSO) to a stock solution (10 mM) and kept at − 80 °C. The working solution was freshly prepared before being added to the culture medium. The final DMSO concentration was lower than 0.1% for in vitro co-incubation. For the CGA treatment group, the CGA-containing medium was replaced by a fresh culture medium after the indicated hours of incubation. For the co-treatment group, the CGA-containing medium was added 2 hours before RT.

### 2.3. Assessment of Cell Viability

Cell viability was determined using the Cell Counting Kit-8 (Beyotime, Shanghai, China) according to the manufacturers' instructions. Briefly, 5 × 10^3^ cells were seeded in a well of 96-well flat-bottomed plate and incubated for 24 h and then placed in serum-starved conditions for a further six hours. Subsequently, different concentrations of CGA were added to treat the cells for 24 h, and then 10% CCK-8 dye was added to each well maintained for another 1.5 h. Viable cells were detected by measuring the absorbance at 450 nm by a microplate reader (BioTek ELx800, USA). Every experiment was run in sextuplicate and performed for three times [26].

### 2.4. Clonogenic Assays

Clonogenic survival analysis was conducted as previously described [27, 28]. The colonies containing more than 50 individual cells are counted.

### 2.5. Radiation Therapy

RT was delivered using a RS-2000 Biological Irradiator (Rad source, Alpharetta) at a dose rate of 2 Gy/minute as previously described. Focal irradiation was delivered to inoculated tumors via lead shielding [29].

### 2.6. Measurement of Intracellular ROS

Total intracellular ROS was determined by staining cells with dichlorofluorescein diacetate (DCFH-DA, Beyotime) [30]. After pretreatment with CGA for 24 h, cells were washed with PBS and incubated with 10 *μ*M DCFH-DA at 37 °C for 30 min. Cells were then washed twice with PBS and analyzed by flow cytometry in 30 minutes (BD, AccuriTM C6). The data were analyzed with FlowJo7.6.1 software (Verity Software House, USA).

### 2.7. Cell Apoptosis Analysis

Cell apoptosis was evaluated by flow cytometry after staining with an annexin V-FITC- PI apoptosis detection kit (Keygen Biotech, Nanjing, China), according to the manufacturer's instructions. Fluorescence was measured using a flow cytometer (FACScan, Becton Dickinson, USA). The data were analyzed with FlowJo7.6.1 software (National Institutes of Health).

### 2.8. Confocal Analysis

Huh7 cells were grown on coverslips and treated with or without CGA, followed by exposure to single irradiation. Twenty-four hours later, cells were fixed in 4% PFA, followed by cell permeabilization with 0.5% Triton X-100 and blocked with 1% BSA and 0.5% goat serum in phosphate-buffered saline (PBS). Cells were incubated with the anti-phospho-Histone H2A.X (S139) or anti-53BP1 antibody overnight, followed by incubation with Alexa Fluor 488 secondary antibody (Boster, BA1127). Slides were mounted using anti-fading reagent with DAPI (Solarbio, S2110) and analyzed using a Leica TCS SPE confocal laser scanning microscope (Leica, Heidelberg, Germany). Fluorescence was excited with a 488 nm line and collected with a 517 nm filter. The LAS AF software (Leica, Heidelberg, Germany) was used for image acquisition.

### 2.9. Nrf2 Translocation

Cells were treated without and with CGA for 0, 0.5, 3, and 6 h. The cytoplasmic and nuclear proteins of each sample were obtained using the Minute™ Cytoplasmic & Nuclear Extraction Kits for Cells from Invent (Inventbiotech, SC-003). 60 *μ*g of each sample was loaded onto a 12-20% SDS-PAGE gel and transferred onto a PVDF membrane. The membranes were incubated with the Nrf2 antibody overnight at 4 °C and were then incubated with the appropriate secondary antibodies at room temperature for one hour. GAPDH and H3 were used as a cytoplasmic and nuclear marker, respectively. Detection was performed using the ECL Western blotting detection system (Thermo Scientific, Rockford, IL). The immunoblot was analyzed with Image J software.

### 2.10. Transient Transfection of Small RNA Interference

The Huh-7 cells were transfected with Nrf2 siRNA synthesized by GenePharma (Shanghai, China) using Lipofectamine® 3000 reagents (Invitrogen, Carlsbad, CA, USA) following the manufacturer's instructions. After 24 h of transfection, the cells were treated with RT, CGA, or combination treatment.

### 2.11. Quantitative Real-Time Polymerase Chain Reaction (PCR) Analysis

Total RNA was extracted by using triazole reagent (Invitrogen, USA) according to the manufacturer's instructions. One-step qRT-PCR was done with TaKaRa One-Step SYBR® PrimeScriptTM PLUS RT-PCR Kit on StepOne real-time PCR machine by *ΔΔ*Ct method. Oligonucleotide primers for Nrf2 (forward, 5′- AAGAATAAAGTCGCCGCCCA -3′; reverse, 5′-AGATACAAGGTGCTGAGCCG-3′) were synthesized by Sangon Biotech (Shanghai, China). Reaction parameters were as follows: step 1, 42 °C for 5 minutes; step 2, 95 °C for 10 seconds; step 3, 95 °C for 10 seconds; step 4, 50 °C for 30 seconds; and step 5, 72 °C for 30 seconds. Step 3 to step 5 was repeated for 35 cycles. The level of Nrf2 mRNA was analyzed by StepOne Software version 2.1. The relative amount of Nrf2 was normalized to the amount of endogenous *β*-actin.

### 2.12. Western Blot Analysis

Protein extraction was performed in Radioimmunoprecipitation assay buffer (RIPA) (Auragene, Changsha, China) and centrifuged at 13000 rpm for 20 min at 4 °C. Protein concentrations were determined using a BCA protein assay kit (Auragene, Changsha, China). Proteins were separated on 4%-20% gels and then blotted onto nitrocellulose membranes and probed with the first antibody, followed by the appropriate secondary antibodies (Boster, Wuhan, China). Immunodetection was accomplished via the ECL plus western blotting detection system (Auragene, Changsha, China). The signal intensity was determined using the Image J software.

### 2.13. Animal Assay

Six-week-old C57BL/6 mice weighing 19-23 g were obtained from the Animal Core Facility of Nanjing Medical University (Nanjing, China) and maintained in laminar flow cabinets under SPF conditions under a 12-h dark/light cycle. The mice were acclimatized for at least one week before the experiment. Hep1-6 cells (5 × 10^5^ cells) were subcutaneously injected into the right flank of the mice. After the tumor reached 50 mm^3^, mice were randomly assigned to four groups. RT was delivered using RS-2000 Biological Irradiator (Rad source, Alpharetta) the day after grouping (8 Gy, twice a week) [31]. For CGA group, CGA (60 mg/kg) dissolved in saline (100 ul) was given by intraperitoneal injection. For the combination group, CGA (60 mg/kg) was intraperitoneally injected 2 hours prior to RT (8 Gy, twice a week). For control and RT treated group, the same volume of saline was injected during the treatment period. All animal experiments are conducted according to the institutional guidelines of the Animal Care Committee (The First Affiliated Hospital, Zhejiang University School of Medicine, Zhejiang, China).

### 2.14. Histology

The liver of mice was dissected after euthanasia, then washed in ice cold PBS and fixed in 10% neutral-buffered formalin for 24 h. Next, tissues were dehydrated using a concentration gradient of alcohol prior to paraffin embedding. Sections of tissues (5 *μ*m) were prepared for staining with hematoxylin and eosin (H&E). Images were captured using a fluorescent microscope (Olympus BX51).

### 2.15. Statistical Analysis

Statistical analyses were performed by Student's *t*-test or one-way analysis of variance using GraphPad Prism 7.0 software (La Jolla, CA, USA). The data shown in the study were obtained in at least three independent experiments, and all results represent the mean ± SEM. Differences with *P* values <0.05 were considered statistically significant.

## 3. Results

### 3.1. Radiation Induced Elevated Apoptosis and Cytotoxicity by Generating ROS in HCC Cells

**Figure 1:**
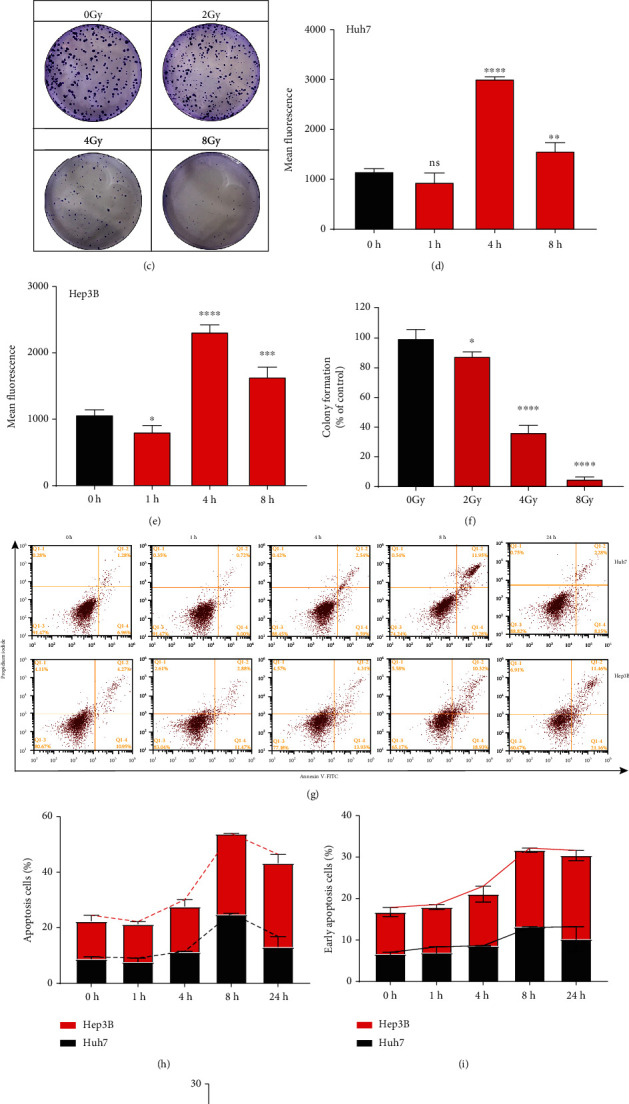
Radiation induced oxidative stress and cytotoxicity in HCC cells. (a), (b) Fluorescence-activated cell sorting (FACS) results of ROS levels in Huh7 and Hep3B cells at the indicated time points after a single dose of 8 Gy as determined using DCFH-DA probes. (c) Colony formation assays in HCC cells with indicated doses of RT. The crystal violet staining of one representative experiment. (d), (e) Quantitation of ROS levels in (a and b). (f) Quantification of colony formation rates of control. (g) Dynamic changes of apoptosis within 24 h after a single dose of 8 Gy in Huh7 and Hep3B cells. (h) Quantitation of apoptosis levels at the indicated time points in (g). (i) Quantitation of early apoptosis levels in at indicated time points in (g). (j) Quantitation of late apoptosis levels in HCCs at the indicated time points at indicated time points in (g). Error bars indicate means ± SEM for three independent experiments. ns (not significant), ∗*P* < 0.05, ∗∗*P* < 0.01, and ∗∗∗*P* < 0.001 comparing with the control group.

We irradiated HCC cells with a single dose of 8 Gy to measure the dynamic changes of oxidative stress after RT. The flow cytometry outcomes showed that ROS levels increased 1 h after RT and then reached a peak within 4 h after RT. Later on, ROS levels began to recover and stayed at relatively high levels compared with those in non-irradiated cells (Figures 1(a), 1(b), 1(d), and 1(e)). Generally, ROS levels in both cell lines showed similar dynamic change patterns. We further checked the apoptosis percentage at a series of time points in HCC cell lines to determine the cytotoxicity of RT. In the Huh7 cell line, the apoptotic cell rate reached a peak in 8 h and recovered to the regular status 24 h after irradiation, as revealed by annexin V and propidium iodide staining (Figures 1(g)–1(i)). Similarly, in the Hep3B cell line, the apoptotic cell rate reached a peak in 8 h and remained high 24 h after RT (Figures 1(g)–1(i)). We also conducted colony formation assays with Huh7 cells and observed a dose-dependent reduction in colony formation abilities (Figures 1(c) and 1(e)). These results suggested that RT induced a quick elevation of ROS and apoptosis level and exhibited dose-dependent cytotoxicity in HCC cells.

### 3.2. CGA Attenuated Radiation-Induced ROS and Apoptosis in HCC Cells Rather in Normal Hepatocytes

**Figure 2:**
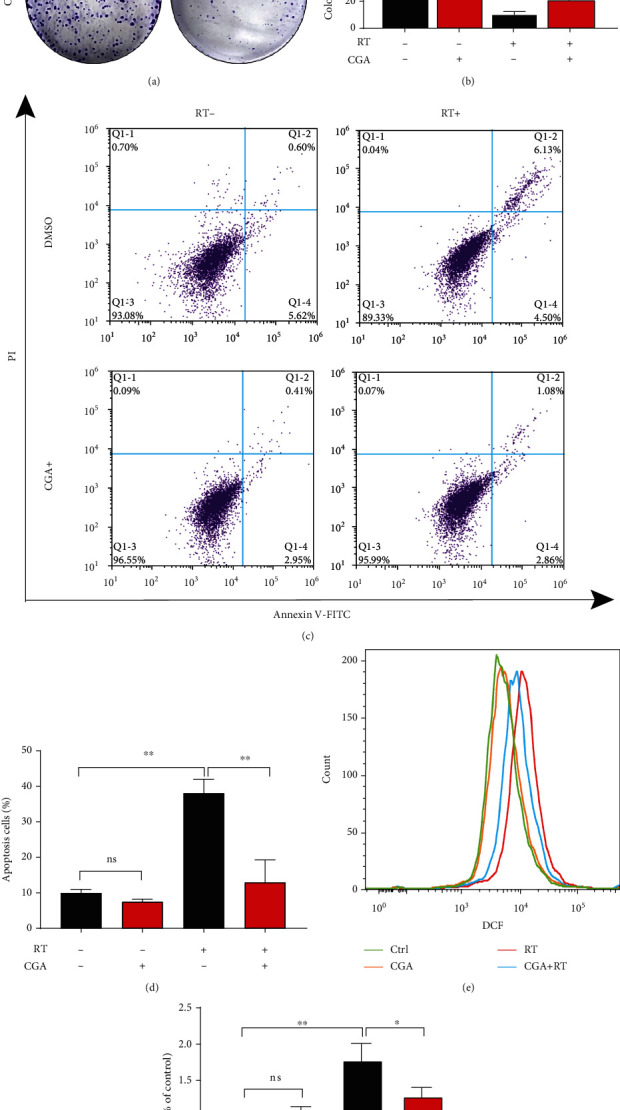
CGA shielded HCC cells from RT-induced cytotoxicity by reducing apoptosis and ROS levels. (a) Colony formation assays with the indicated treatments. Huh7 cells were first pretreated with or without 10 *μ*M CGA for 2 h and then exposed to a single dose of 4Gy or sham irradiation. The crystal violet staining of one representative experiment. (b) Quantification of colony formation rate of control. (c) Flow cytometry of annexin/PI double-stained of control, CGA, RT, and combination treatment. (d) Quantification of the percent of apoptosis cells in indicated groups in (c). (e) Flow cytometry results of ROS levels in Huh7 cells with CGA or RT treatment, combination treatment compared to control. (f) Quantification of ROS levels in indicated groups in (e). Error bars indicate means ± SEM for three independent experiments. ns (not significant), ∗*P* < 0.05, ∗∗*P* < 0.01, and ∗∗∗*P* < 0.001 comparing with the control group.

We treated HCC cell lines (including Huh7 and Hep3B) with various concentrations of CGA to evaluate the cytotoxicity of CGA in vitro. Twenty-four hours later, the cell cytotoxicity was determined using the CCK8 assay. As shown in Figures S1A and S1B, the incubation with CGA at concentrations lower than 100 *μ*M exerted no effect on cell viability. We pretreated cells with CGA or the equivalent volume of DMSO for 2 h before exposing them to RT to test the effects of CGA on irradiated cells (Huh7, Hep3B, and LO2). The results showed that the pretreatment with 10 *μ*M CGA significantly decreased RT-induced cytotoxicity in HCC cell lines, but not in normal hepatocytes LO2 (Figures 2 and S2). The clonogenic survival assay was used to explore the effect of CGA on RT-induced cell death. Compared with the RT treatment, CGA pretreatment significantly increased the number of colonies after RT (Figures 2(a) and 2(b)). Furthermore, the intracellular ROS level and apoptosis were measured by flow cytometry to investigate whether oxidative stress was involved in the cytoprotective effectiveness of CGA. Based on our understanding of the dynamic changes of oxidative stress induced by RT in Figure 1, we collected cells 4 h and 8 h after RT to test their levels of ROS and apoptosis. As shown in Figures 2(c) and 2(d), pretreatment with 10 *μ*M CGA for 2 h significantly decreased the level of apoptosis after exposure to 8 Gy irradiation. Similarly, a marked reduction of the ROS level (*P* < 0.05) was observed in the CGA pretreatment group, as shown in Figures 2(e) and 2(f) (*P* < 0.01). However, in LO2 cells, there was no significant difference in ROS level between the irradiation group and CGA pretreatment group (Figure S2A). As shown in supplemental Figure 2(b), LO2 cells were first pretreated with or without 10 *μ*M CGA for 2 h and then exposed to a single dose of 0, 8, 10, and 12 Gy or sham irradiation; CGA pretreatment group showed no significantly increased in cell viability compared with the corresponding no-CGA treated groups. These results indicated that CGA, as a nontoxic antioxidant, could attenuate cytotoxicity induced by RT via reducing apoptosis and ROS levels in HCC cells.

### 3.3. CGA Decreased Radiation-Induced DNA Damage

**Figure 3:**
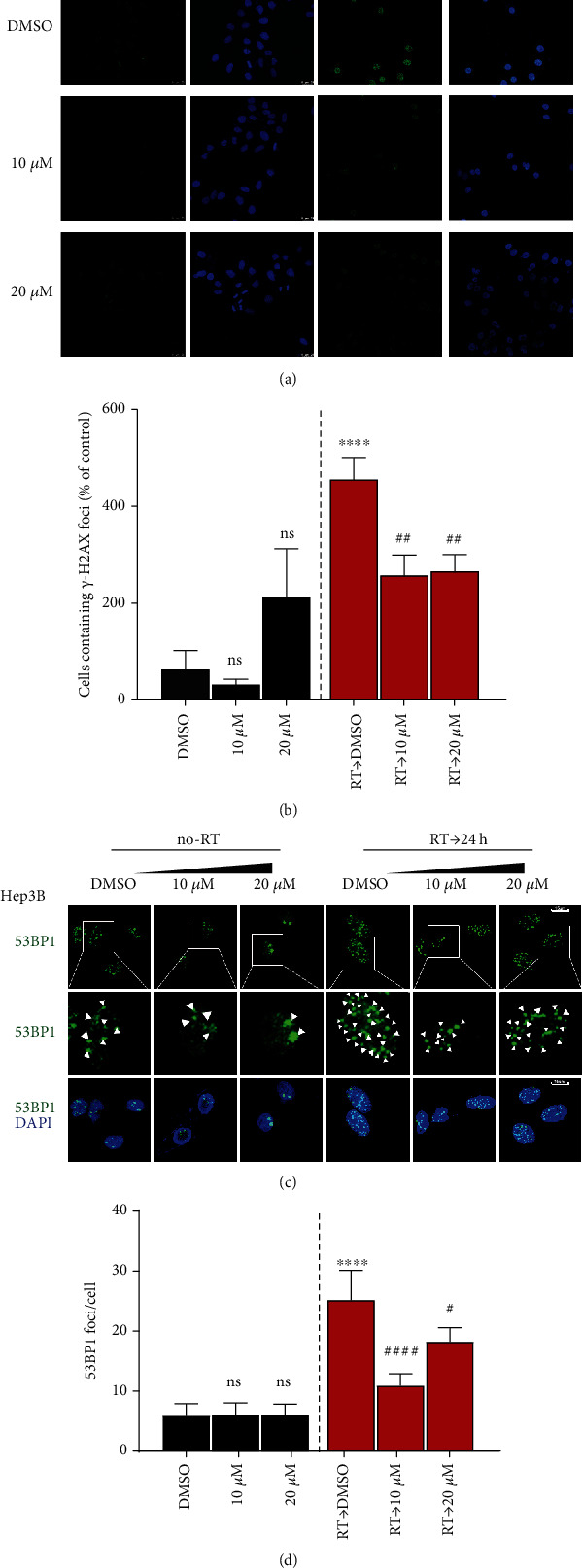
CGA decreased RT-induced DNA damage. (a), (b) *γ*-H2AX cell staining and quantification of the percentage of *γ*-H2AX positive cells in DMSO group, low and high CGA concentration group, RT group, and combination treatment group. (c), (d) 53BP1 staining and quantification of 53BP1 foci in cells in DMSO group, low and high CGA concentration group, RT group, and combination treatment group. Error bars indicate means ± SEM for three independent experiments. ns (not significant), ∗*P* < 0.05, ∗∗*P* < 0.01, and ∗∗∗*P* < 0.001 comparing with the control group.

Since RT functions mainly through DNA damage, we further explored whether CGA-mediated increase in survival was due to decreased DNA damage rates. The immunofluorescence staining of *γ*-H2AX and 53BP1 foci were used to determine the effect of CGA on DNA damage because they were crucial mediator proteins involved in DNA damage [32]. We found that the percentage of *γ*-H2AX-positive cells increased rapidly and massively after irradiation and showed a significant decrease after CGA pretreatment, no matter at high or low concentrations (Figures 3(a) and 3(b)). Besides, we also calculated 53BP1 foci numbers in HCC cells after DMSO, CGA, RT, and combination treatment. As shown in Figures 3(c) and 3(d), the number of 53BP1 foci rapidly increased after RT, while only a few foci were present in cells without RT. In line with what we observed in the detection of *γ*-H2AX foci, the cells pretreated with CGA before RT showed less 53BP1 foci sustained after 24 h than DMSO-treated cells (Figures 3(c) and 3(d)). These results suggested that CGA could efficiently reduce RT-induced DNA damage, whether at a high or a low concentration.

### 3.4. CGA Suppressed Radiation-Induced Cell Damage via Nrf2 Activation

**Figure 4:**
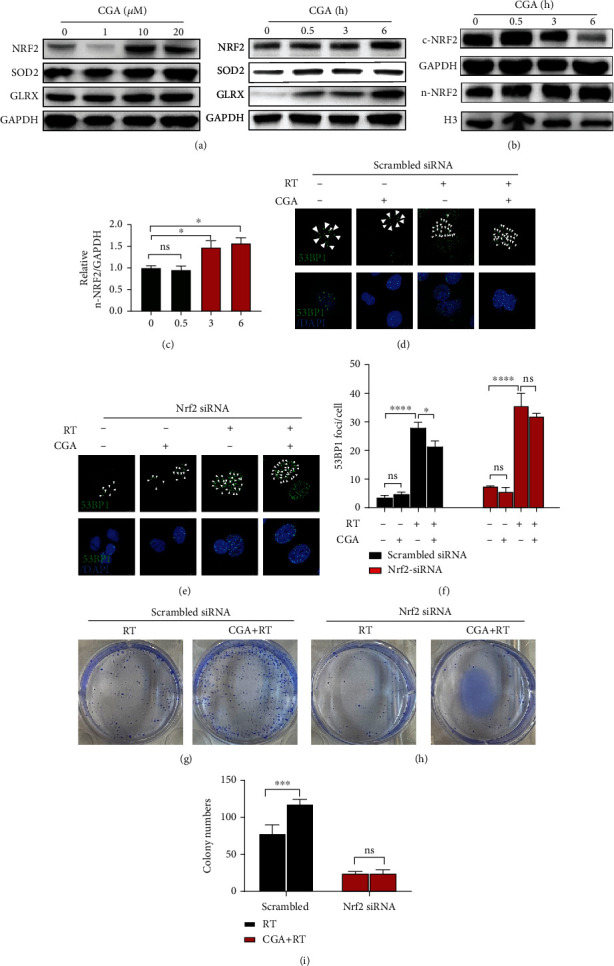
Involvement of Nrf2 signaling in the radioresistance effect of CGA. (a) The protein expression levels of Nrf2, SOD2, and GLRX after incubation with indicated concentrations of CGA (0, 1, 10, and 20 *μ*M) or with 10 *μ*M CGA for indicated time (0, 0.5, 3, 6 h). (b) Cytoplasmic and nuclear expression of Nrf2 after incubation with CGA for indicated hours (0, 0.5, 3, and 6 h) and (c) quantification of relative nuclear expression of Nrf2. (d, e) Representative images of 53BP1 staining in HCC cells transfected with scrambled siRNA or Nrf2 siRNA under DMSO, CGA or RT treatment, and combination treatment. (f) Quantification of 53BP1 foci in HCC cells transfected with scrambled or Nrf2 siRNA, respectively. (g, h) Colony formation assay after a single dose of 4Gy or combination of CGA with a single dose of 4Gy treatment in cells transfected with scrambled siRNA or Nrf2 siRNA. (i) Quantification of colony numbers in cells transfected with scrambled or Nrf2 siRNA, respectively. Error bars indicate means ± SEM for three independent experiments. ns (not significant), ∗*P* < 0.05, ∗∗*P* < 0.01, and ∗∗∗*P* < 0.001 comparing with the control group.

With the help of a compound-gene interaction database and online bioinformatics tools, we found that Nrf2 might be the key target of CGA (Figure S3). We treated HCC cells with a series of concentrations of CGA (1, 10, and 20 *μ*M) for 24 h. The protein expression levels of Nrf2 and its target genes SOD2 and GLRX significantly increased in the presence of CGA (Figure 4(a)). We also found that Nrf2 and its target genes were induced time-dependently (right panel in Figure 4(a)). We extracted cytoplasmic and nuclear proteins at indicated hours (0, 0.5, 3, and 6 h) after CGA treatment to identify nuclear translocation. In the cytoplasm, Nrf2 expression slowly decreased as more Nrf2 moved into the nucleus (Figures 4(b) and 4(c)). These results indicated that CGA also enhanced the nuclear translocation of Nrf2. Next, we used siRNA to confirm whether CGA conferred radioresistance by inducing Nrf2 activation. Nrf2 knockdown efficiency was verified by quantitative polymerase chain reaction and Western blot analysis, as shown in Figure S4. As expected, Nrf2 knockdown abolished the CGA-mediated reduction in RT-induced DNA damage (Figures 4(d)–4(f)). We also tested whether Nrf2 knockdown affected the colony formation ability of HCC cells. We plated the same number of cells transfected with scrambled-siRNA or Nrf2-siRNA before irradiation. In Nrf2 siRNA–transfected cells, CGA-mediated radiotherapy resistance disappeared (Figures 4(g)–4(i)). These data verified that the primary radioresistance mechanism of CGA was through Nrf2 activation and Nrf2 knockdown blocked the CGA-conferred radioresistance effects in HCC cells.

### 3.5. CGA Reduced Radiation-Induced Tumor Inhibition in HCC Xenografts

**Figure 5:**
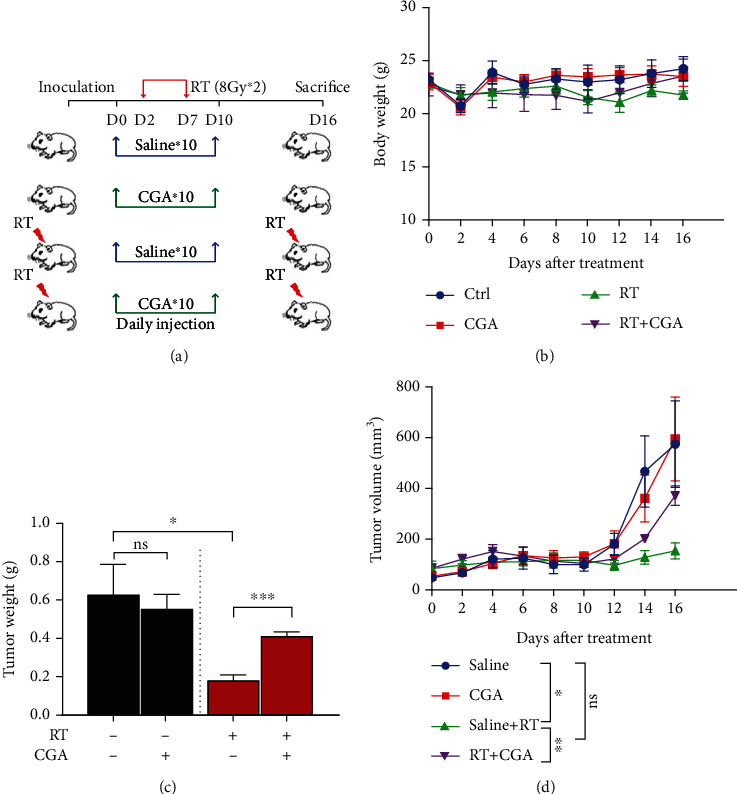
CGA conferred radioresistance of HCC in vivo. (a) The treatment schedule was illustrated in the diagram. (b) Body weight of mice in four indicated groups of mice. (c) Weight of dissected tumors from mice in saline, CGA (60 mg/kg), saline plus RT, and CGA plus RT treatment groups. (d) Tumor growth curves of xenografts in four indicated groups of mice. Values are the mean ± SEM, *n* = 6/group; ∗*P* < 0.05, ∗∗*P* < 0.01, and ∗∗∗*P* < 0.001 comparing with the control group.

We injected Hep1-6 cells subcutaneously into the right leg and exposed xenografts to saline treatment, CGA treatment, RT treatment, and CGA + RT treatment to examine the suppression of radiation-induced tumor inhibition by CGA *in vivo*. The experimental design is schematized in Figure 5(a). No significant difference in body weight was found among the four groups (Figure 5(b)). The tumor weight of mice in the RT group decreased significantly, while no significant difference was observed in the volume of mice in the CGA + RT group compared with the control group (Figure 5(c)). The combined treatment group showed a significant gain of tumor volume compared with the RT group, implying a CGA-conferred radio-resistant effect (Figure 5(d)). Consistent with *ex vivo* results, CGA suppressed radiation-induced tumor inhibition in the HCC mouse model. In addition, we also found there were no significant difference in liver morphology and serum ALT, AST, BUN, and LDH level between normal mice and CGA treated, RT treated, and CGA + RT treated mice (Supplemental Figures 5 and 6). The above data suggest that CGA may have limited effect during irradiation in mice normal liver.

## 4. Discussion

**Figure 6:**
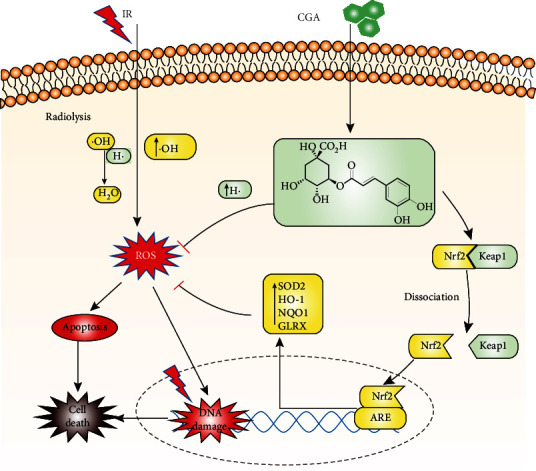
Mechanisms of radiation resistance by CGA. CGA is able to neutralize free radicals that are produced by RT. Furthermore, it activates Nrf2/ARE signaling pathways, leading to increased detoxification ability. CGA, via inhibition of oxidative stress and alleviate DNA damage, improves HCC genomic stability, thereby reducing the therapeutic effect of RT.

This study was novel in demonstrating that CGA, previously regarded as a chemopreventive drug in several types of cancer, might confer radioresistance in HCC through modulating the ROS/Nrf2 signaling pathway. The schematic diagram of the mechanism is summarized in Figure 6.

HCC is a leading cause of cancer-related death worldwide and is a major health problem, especially in developing countries [3]. RT plays an important role in the treatment and survival of patients with HCC, but the efficacy is limited in some patients manifested as radiation resistance. RT triggers DNA damage, for the most part through the generation of free radicals that cause a variety of DNA lesions. Antioxidant agents capable of removing free radicals and activating the Nrf2 antioxidant pathway have been reported to have the radioprotective ability in fibroblasts and bone marrow hematopoietic cells [15, 33]. However, whether antioxidants affect the therapeutic effect of RT on tumors is rarely elucidated.

CGA, a major polyphenol compound with robust antioxidant ability, exists naturally in various agricultural products such as coffee and tea [16]. So far, CGA has been engaged in 16 clinical trials, including treatment for diabetes, dyslipidemia, metabolic syndrome, endothelial dysfunction, and overweight conditions, in addition to six cancer treatment trials. No clinical trial of the use of CGA in liver cancer has been conducted yet. Previous laboratory studies have found direct free radical scavenging activity and anti-HCC ability of CGA [21, 23]. The *in vitro* and *in vivo* models were established in our study to investigate the effects of CGA during RT treatment on HCC.

First, we tested whether CGA incubation alone exerted cytotoxicity effects on HCC cells; we found it nontoxic within a concentration of 100 *μ*M in several HCC cell lines (Huh7 and Hep3B). In addition, we found that HCC cells were quite sensitive to RT *in vitro*. A serial of doses of RT from 2 to 6 Gy inhibited clonogenic survival by more than 70% (*P* < 0.0001). Next, we pretreated HCC cells with CGA for 2 h and then exposed them to RT. Compared with the control treatment, CGA preincubation significantly increased the survival rate of HCC cells after RT (*P* < 0.05). Subsequently, we observed the dynamic changes of oxidative stress induced by RT [1] and found that ROS and apoptosis reached a peak within 8 h. In the control group, CGA did not change the basal ROS level and apoptosis percentage of HCCs. However, pretreatment with CGA at noncytotoxic concentrations significantly eliminated the rapid and robust increase in ROS and apoptosis induced by RT. These results were in accordance with the previously established antioxidant properties of CGA [18, 23, 34, 35]. However, it is interesting that these phenomena were not observed in normal hepatocyctes LO2 cells (Supplemental Figure 2). Besides, we also studied the effect of CGA on RT-induced DNA damage. Compounds with antioxidant characteristics have been shown to exert protective effects against DNA damage [14]. Also, under CGA pretreatment, the expression of *γ*-H2AX and 53BP1 decreased after RT.

Since RT is linked to ROS generation and ROS levels are mainly mediated by the Nrf2 signaling pathway [8, 36], we proposed that CGA might counteract the effects of RT by removing radicals and activating the Nrf2 signaling pathway. To verify our hypothesis, we treated cells with different doses of CGA and extracted cytoplasmic and nuclear proteins from CGA-treated cells. The net effect included activated Nrf2 and its downstream target genes and decreased ROS levels. Furthermore, the blockade of the Nrf2 signaling pathway with Nrf2 siRNA reversed the aforementioned CGA-mediated decrease in RT-triggered DNA damage and growth inhibition. Finally, we explored whether CGA affected the efficacy of RT *in vivo*. We found that the additional administration of CGA reduced radiation-induced HCC growth inhibition by decreasing tumor apoptosis. Previously, Yan et al. [21] reported results opposite to ours that CGA could prevent the progression of HCC in HepG2 (p53 wild-type) cells derived from xenograft nude mice. However, we used p53 mutant Huh7 cells in animal models, which suggested that p53 might play an important role in the CGA-mediated prevention of HCC. Further exploration is underway to elucidate the influence of p53 loss on CGA-mediated radioresistance.

In conclusion, this study provided *in vitro* and *in vivo* evidence that CGA hindered the treatment efficacy of RT on HCC. *In vitro*, CGA inhibited RT-induced ROS generation, apoptosis, and DNA damage. *In vivo*, CGA promoted HCC growth after RT treatment. The possible molecular mechanism involved ROS scavenging, Nrf2 nuclear translocation, and downstream signaling pathway activation.

## Data Availability

All data generated or analyzed in this study are included in the manuscript and supplemental materials.

## Abbreviations

ARE: Antioxidant response element
CGA: Chlorogenic acid
DCFH-DA: Dichlorofluorescein diacetate
HCC: Hepatocellular carcinoma
HO-1: Heme oxygenase-1
NQO-1: NAD(P)H:quinone oxidoreductase-1
Nrf2: Nuclear factor erythroid 2-related factor 2
ROS: Reactive oxygen species
RT: Radiation therapy.

## Supplementary Materials

Supplementary Materials Methods and Materials. (1) Antibodies. (2) Quantitative real-time polymerase chain reaction (PCR) analysis. (3) Si-RNA sequence. Supplementary figures and legends. Figure S1. CGA alone conferred no cytotoxicity on HCC cells. Figure S2. CGA did not shield LO2 cells from RT-induced cytotoxicity by reducing IR-induced ROS levels. Figure S3. Genes interacting with chlorogenic acid and protein interaction network analysis. Figure S4. Verification of Nrf2 knockout efficiency. Figure S5. Liver H&E staining. Figure S6. The serum ALT, AST, BUN, and LDH levels of the mice in different groups.
