# Supplementary material for: Chlorogenic Acid, the Main Antioxidant in Coffee, Reduces Radiation-Induced Apoptosis and DNA Damage via NF-E2-Related Factor 2 (Nrf2) Activation in Hepatocellular Carcinoma
Source: Oxid Med Cell Longev. 2022 Aug 2;2022:4566949. doi: 10.1155/2022/4566949 (PMC9363170; doi:10.1155/2022/4566949)
Supplement: Supplementary Materials — Methods and Materials. (1) Antibodies. (2) Quantitative real-time polymerase chain reaction (PCR) analysis. (3) Si-RNA sequence. Supplementary figures and legends. Figure S1. CGA alone conferred no cytotoxicity on HCC cells. Figure S2. CGA did not shield LO2 cells from RT-induced cytotoxicity by reducing IR-induced ROS levels. Figure S3. Genes interacting with chlorogenic acid and protein interaction network analysis. Figure S4. Verification of Nrf2 knockout efficiency. Figure S5. Liver H&E staining. Figure S6. The serum ALT, AST, BUN, and LDH levels of the mice in different groups. [file 4566949.f1.docx]

**Supplemental materials**

**Methods and Materials**

1. Antibodies:

| Antibodies | **Company** |
| --- | --- |
| anti-Nrf2 | Cell Signaling, #12721; Boster, BM4842 |
| anti-GAPDH | Santa Cruz, sc-32233 |
| anti-Glutaredoxin 1 (GLRX) | Abcam, ab45953 |
| anti-SOD2 | Cell Signaling, #13141 |
| anti-53BP1 | Cell Signaling, #88439 |
| Phospho-Histone H2A.X (Ser139) | Cell Signaling, #2577 |
| anti-b-actin | Abcam, ab32572 |
| Goat Anti-Rabbit IgG DyLight 488 | Boster, BA1127 |
| Goat Anti-Rabbit IgG TRITC | Boster, BA1090 |
| Anti-rabbit IgG, HRP-linked Antibody | Cell Signaling,#7074 |
| Anti-mouse IgG, HRP-linked Antibody | Cell signaling, #7076 |

1. *Quantitative real-time polymerase chain reaction (PCR) analysis*

Oligonucleotide primers for Nrf2 (forward, 5′- AAGAATAAAGTCGCCGCCCA -3′; reverse, 5′-AGATACAAGGTGCTGAGCCG-3′) were synthesized by Sangon Biotech (Shanghai, China). Reaction parameters were step 1: 42℃ for 5minutes, step 2: 95℃ for 10 seconds, step 3: 95℃ for 10 seconds, step 4: 50℃ for 30 seconds, step 5: 72℃ for 30 seconds. Step 3 to step 5 was repeated for 35 cycles. The level of Nrf2 mRNA was analyzed by StepOne Software version2.1. The relative amount of Nrf2 was normalized to the amount of endogenous b-actin.

1. *Si-RNA Sequence*

| **Si-RNA Sequence** |  | **Sequence** |
| --- | --- | --- |
| Negative control | Sense | 5'-UUCUCCGAACGUGUCACGUTT-3’ |
| Negative control | Antisense | 5'-ACGUGACACGUUCGGAGAATT-3’ |
| Si-Nrf2-2 | Sense | 5'-GCCCAUUGAUGUUUCUGAUTT-3’ |
| Si-Nrf2-2 | Antisense | 5'- AUCAGAAACAUCAAUGGGCT T-3' |

**Supplemetal figures &legends**

**
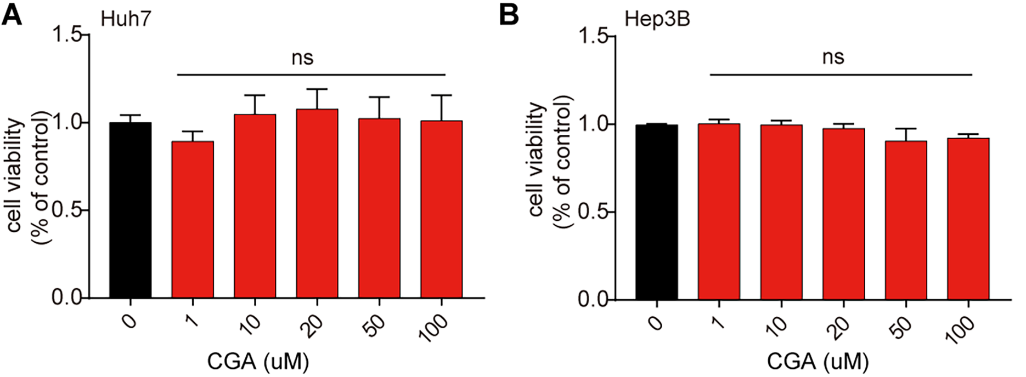
Figure S1. CGA alone conferred no cytotoxicity on HCC cells**

(a) Cell viability from different concentrations of (DMSO-treated), CGA treated (1, 10, 20, 50 and 100 µM) Huh7 cells were measured 48h after incubation; (b) Cell viability from different concentrations of control (DMSO-treated), CGA treated (1, 10, 20, 50 and 100 µM) Hep3B cells was measured 48h after incubation. Error bars indicate means ±SEM for three independent experiments. ns (not significant)

**Figure S2. CGA did not shield LO2 cells from RT-induced cytotoxicity by reducing IR-induced ROS levels.**

**
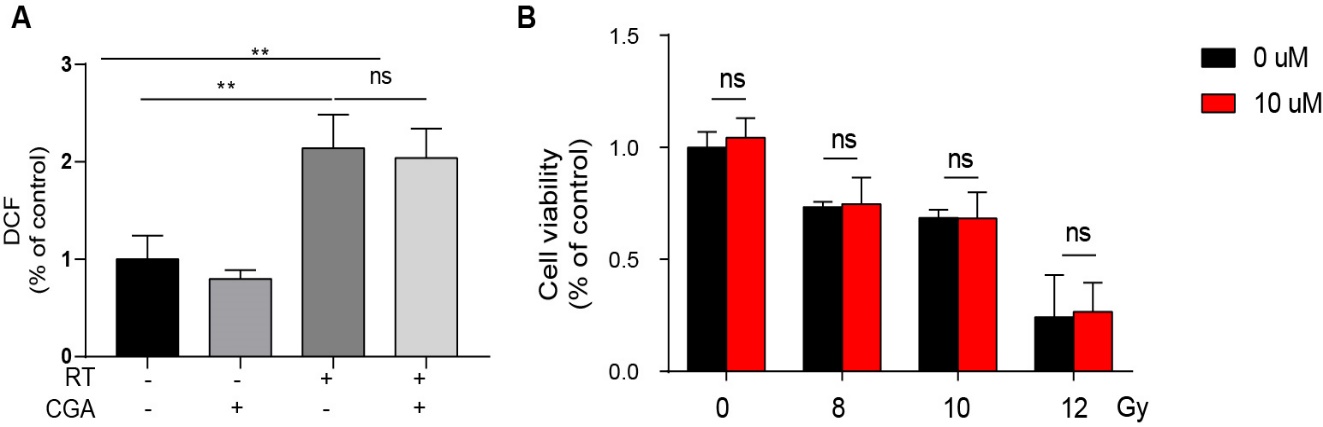
**

（a）LO2 cells were first pretreated with or without 10µM CGA for 2h and then exposed to a single dose of 8Gy or sham irradiation. Flow cytometry results of ROS levels in LO2 cells with CGA or RT treatment, combination treatment compared to control; (b) LO2 cells were first pretreated with or without 10µM CGA for 2h and then exposed to a single dose of 0, 8, 10, 12Gy or sham irradiation. 24h later, the cell viability was tested by CCK-8 staining. Error bars indicate means ±SEM for three independent experiments. ns (not significant), *P<0.05, **P<0.01, ***P<0.001 comparing with the control group.

**Figure S3. Genes interacting with chlorogenic acid and protein interaction network analysis**

**
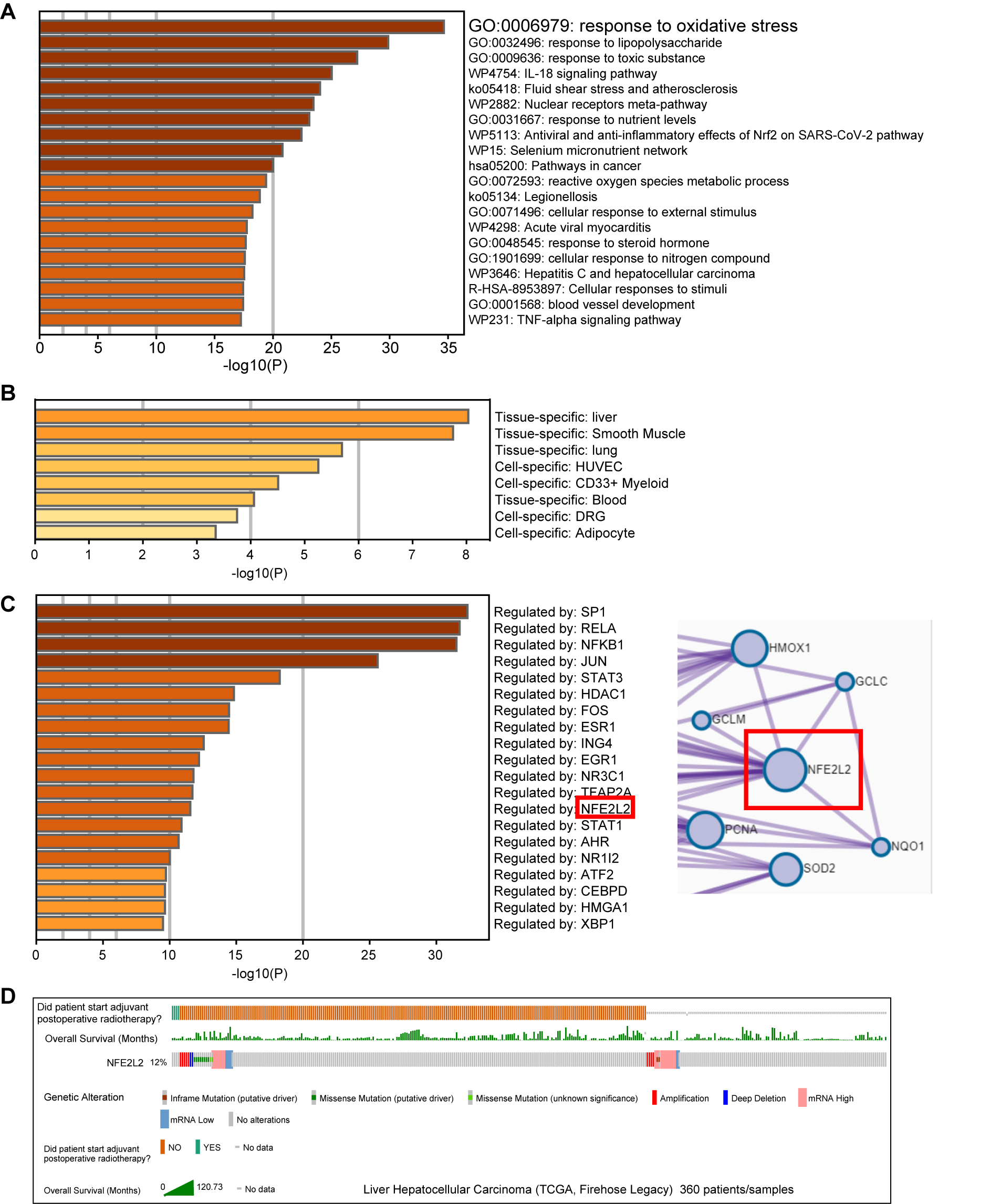
**

(a)176 records of genes interacting with chlorogenic acid were downloaded from the public compound database in NCBI database and uploaded to metascape gene function annotation analysis platform for biological pathway enrichment analysis, organ specific enrichment analysis, (b) protein interaction network analysis and (c) key regulatory molecule analysis (the key gene related to oxidative stress pathway in the red box, NFE2L2, alias of Nrf2).

**Figure S4. Verification of Nrf2 knockout efficiency**


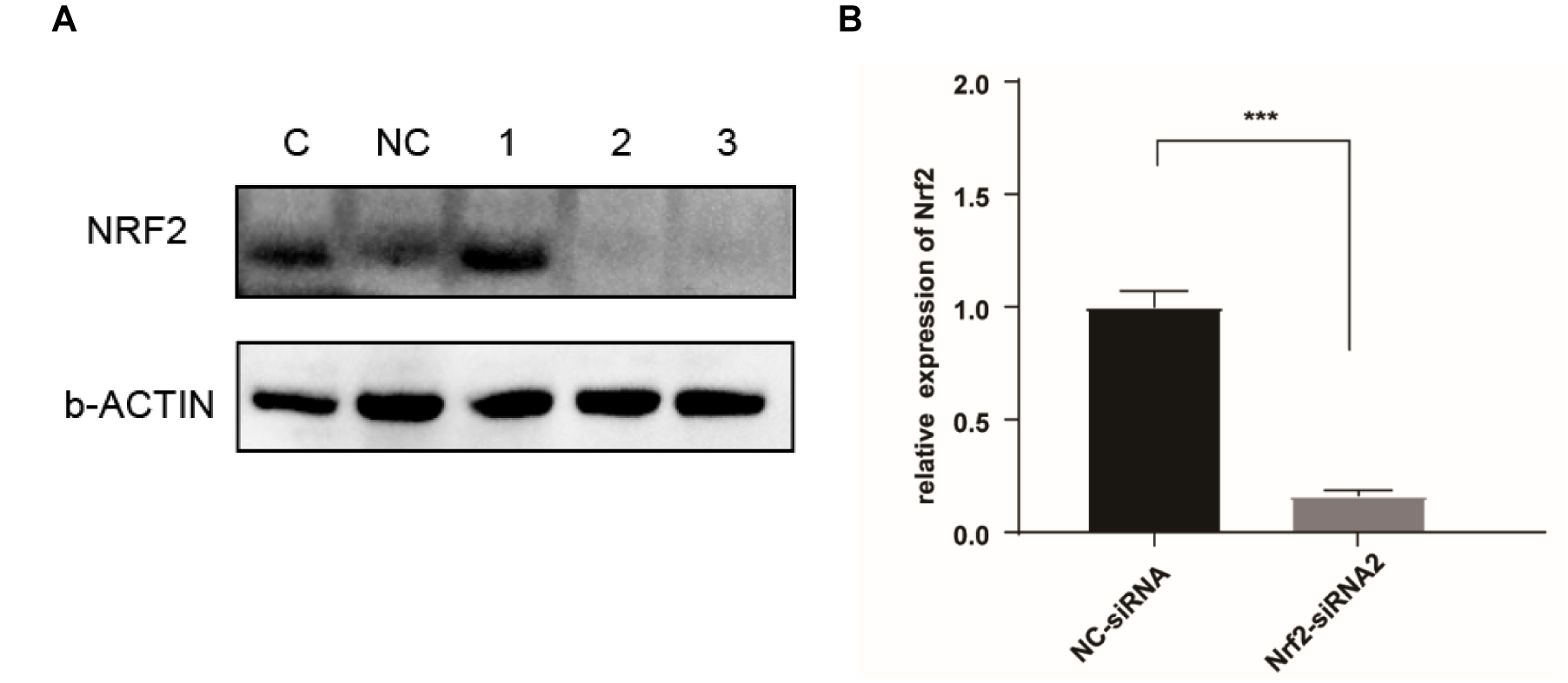
(a)Protein expression of Nrf2 in ctrl cells and cells transfected with negative ctrl siRNA (NC) or Nrf2 siRNAs (1, 2, 3); (b) mRNA level of Nrf2 in cells transfected with NC-siRNA or Nrf2-siRNA2 . Error bars indicate means ±SEM for three independent experiments. ns (not significant), ***P<0.001 comparing with the control group.

**Figure S5. Liver H&E staining**

**
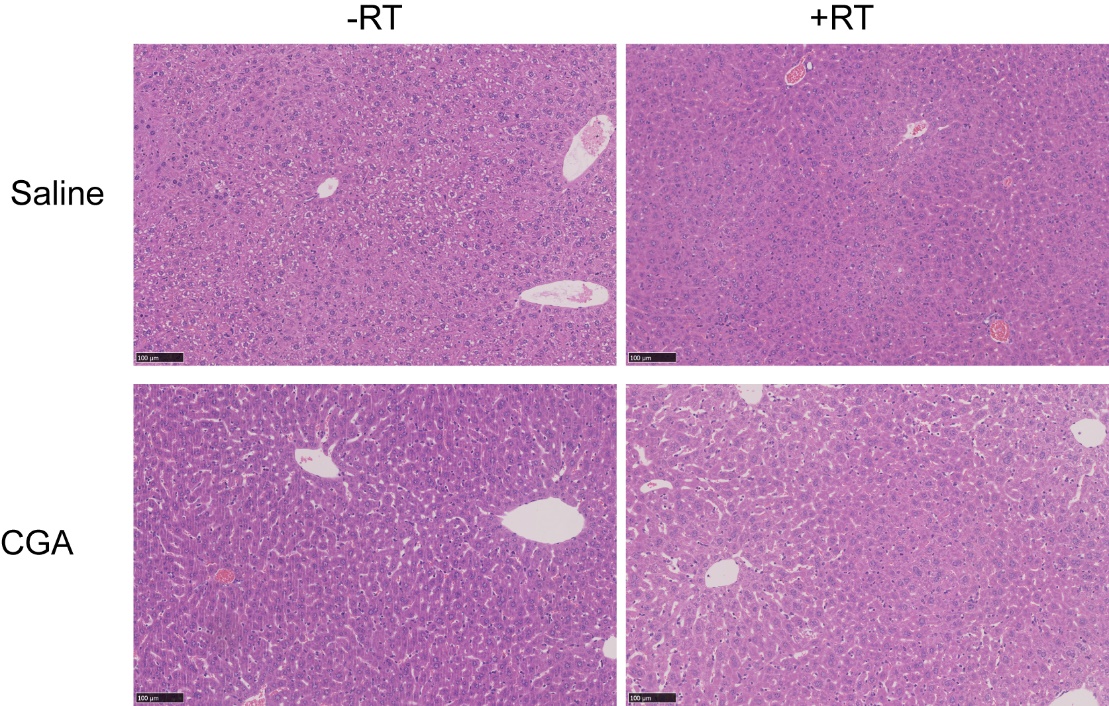
**

**Figure S6. The serum ALT, AST, BUN, LDH levels of the mice in different groups**

**
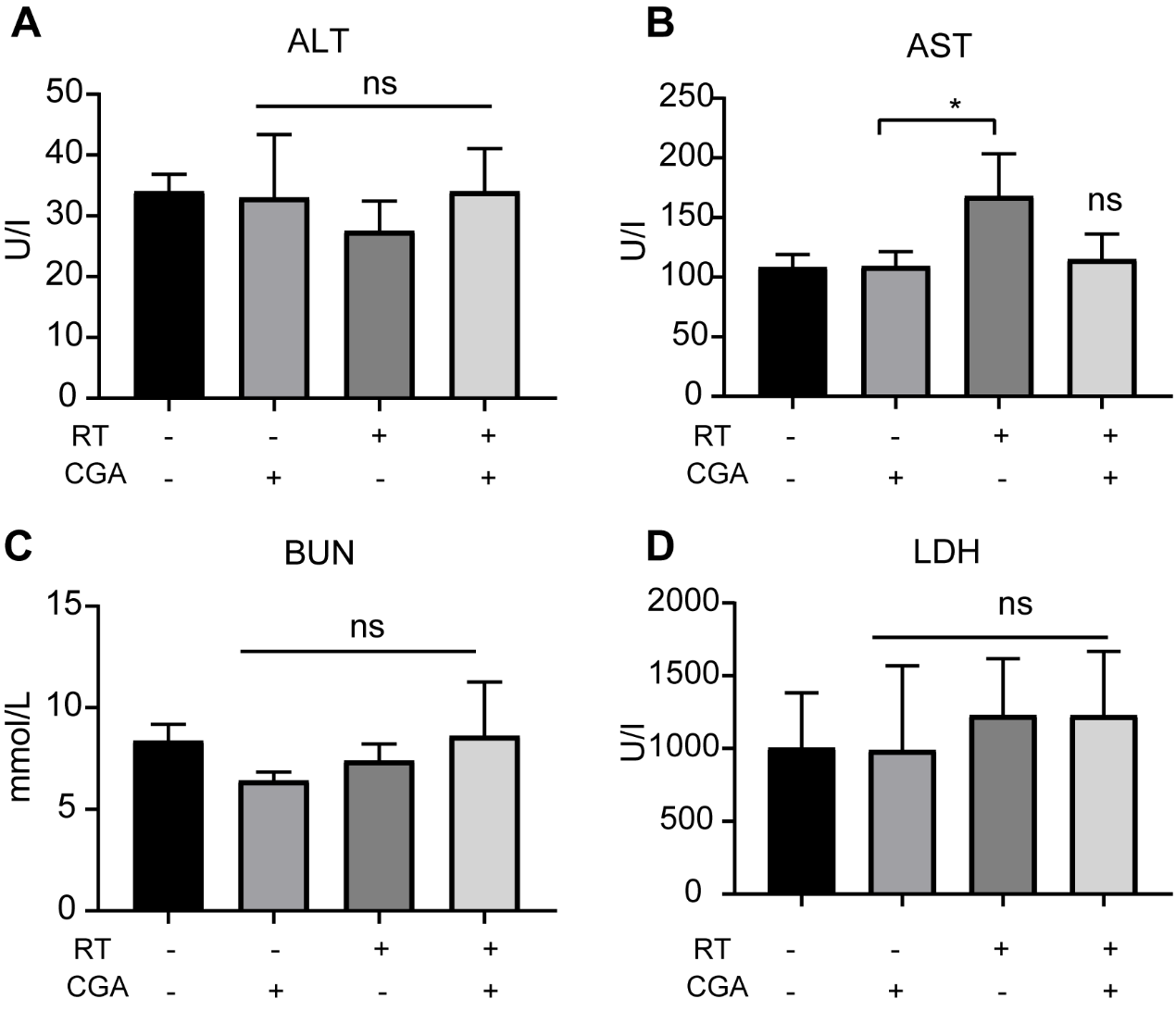
**

The serum (a) ALT, (b) AST, (c) BUN, (d) LDH levels of the mice in different groups (normal mice, CGA-treated mice, irradiated mice and CGA+ irradiated mice). Error bars indicate means ±SEM for three independent experiments. ns (not significant), *P<0.05.
